# Factors Associated with Not Receiving a Booster Dose of COVID-19 Vaccine in Peru

**DOI:** 10.3390/vaccines10081183

**Published:** 2022-07-26

**Authors:** Guido Bendezu-Quispe, Brenda Caira-Chuquineyra, Daniel Fernandez-Guzman, Diego Urrunaga-Pastor, Percy Herrera-Añazco, Vicente A. Benites-Zapata

**Affiliations:** 1 Escuela de Medicina, Universidad César Vallejo, Trujillo 13001, Peru; gbendezuqu@ucvvirtual.edu.pe; 2 Facultad de Medicina, Universidad Nacional de San Agustín, Arequipa 04001, Peru; bcaira@unsa.edu.pe; 3 Escuela Profesional de Medicina Humana, Universidad Nacional de San Antonio Abad del Cusco, Cusco 08000, Peru; 130414@unsaac.edu.pe; 4 Facultad de Ciencias de la Salud, Universidad Científica del Sur, Lima 15067, Peru; 5 Red Internacional en Salud Colectiva y Salud Intercultural, Mexico City 56690, Mexico; percy.herrera@upsjb.edu.pe (P.H.-A.); vbenites@usil.edu.pe (V.A.B.-Z.); 6 Instituto de Evaluación de Tecnologías en Salud e Investigación—IETSI, EsSalud, Lima 14072, Peru; 7 Escuela de Enfermería, Universidad Privada San Juan Bautista, Lima 15067, Peru; 8 Unidad para la Generación y Síntesis de Evidencias en Salud, Universidad San Ignacio de Loyola (USIL), Lima 15012, Peru

**Keywords:** COVID-19 vaccine booster shot, SARS-CoV-2, COVID-19, vaccination refusal, Peru

## Abstract

To determine the factors associated with not receiving the booster dose for COVID-19 in Peru, a cross-sectional study by secondary analysis of a University of Maryland and Facebook survey database assessing the global impact of COVID-19 was conducted. Data of Peruvian users of this social network over 18 years of age who answered the survey between 13 February 2022 and 14 April 2022 were analyzed. We evaluated the association between sociodemographic characteristics, comorbidities, and history of COVID-19 with having received a booster dose for COVID-19. Crude (cPR) and adjusted (aPR) prevalence ratios with their respective 95% confidence intervals (95%CI) were calculated. A sample of 20,814 adults, 21.5% of whom reported not receiving the booster dose, was analyzed. People under 75 years of age had a higher prevalence of not having received the booster dose. Likewise, having a university education (aPR = 1.03; 95%CI: 1.02–1.05), secondary, or pre-university education (aPR = 1.07; 95%CI: 1.05–1.09), or having a primary level or less (aPR = 1.11; 95%CI: 1.05–1.18), were associated with a higher prevalence of not receiving the booster, compared to individuals with a postgraduate education. Being employed (aPR = 1.01; 95%CI: 1.00–1.02), having had COVID-19 (aPR = 1.03; 95%CI: 1.01–1.04) and living in a town (aPR = 1.05; 95%CI: 1.02–1.07) or in a rural area (aPR = 1.06; 95%CI: 1.03–1.10), compared to living in the city, had a similar association. On the contrary, the female gender was associated with a lower prevalence of not receiving the booster (aPR = 0.97; 95%CI: 0.96–0.99). Sociodemographic characteristics and a history of having had COVID-19 were associated with the probability of not having received the booster dose for COVID-19 in the Peruvian population.

## 1. Introduction

The COVID-19 pandemic has affected the world population, and by 2 May 2022, more than half a billion cases (514,027,902) and more than six million deaths (6,237,399) have been reported worldwide due to this disease [1].

Vaccination is one of the public health interventions with the greatest impact on populations, with around five million deaths being prevented annually by immunization schemes [2,3]. During the COVID-19 pandemic, the development and application of vaccination schemes for this disease have been one of the pillars for reducing the transmissibility of SARS-CoV-2 and has been introduced in almost all the countries of the world [2]. Thus, as of May 2022, more than 11 billion doses of the COVID-19 vaccine have been administered worldwide [1], and it is described that COVID-19 vaccines saved nearly 20 million lives worldwide during the first year they were introduced [4].

Some studies have described a progressive reduction in the effectiveness of the primary vaccination schedule for COVID-19, especially in older adults [5], with the immunity and clinical protection provided by the vaccination decreasing below an index considered sufficient at the population level over time [6]. Additionally, the appearance of new variants raises the need for booster doses in patients who completed the primary regimens against SARS-CoV-2 [7,8]. For this reason, to date, campaigns to apply a booster dose of the COVID-19 vaccine have begun in 120 countries [6]. A booster dose is administered to individuals who have completed the primary vaccination schedule that includes schedules of one to two doses depending on the vaccine used. These doses optimize or enhance immune response to a sufficient level of efficacy against the disease with an acceptable safety profile [6,9,10,11,12].

Peru, a country with a population of 33 million, was one of the countries with the highest burden of disease due to COVID-19 worldwide, and by 24 April 2022, more than one million cases and more than two hundred thousand deaths were reported [13]. As in other countries, by 8 May 2022, the vaccination campaign had achieved the administration of more than 72 million doses of the vaccine against COVID-19, with booster dose coverage of 57.3% of the population over 12 years of age [14], which is insufficient to ensure population protection against the disease. In Peru, since November 2021, people aged 18 and over who have received the second dose of the COVID-19 vaccine more than five months ago have been able to receive the booster dose [15]. Several studies have reported factors associated with the intention to receive a booster dose, identifying population groups with less intention, which would serve to guide government dissemination campaigns to improve coverage. However, to the best of our knowledge, no study has evaluated this aspect in comparison with the population that received the booster dose. Since the coverage of the booster dose is still insufficient in our country [14], identifying these groups will allow optimizing resources and directing efforts to improve coverage. Therefore, the objective of this study was to identify the factors associated with not having received the booster dose for COVID-19 in the Peruvian population.

## 2. Materials and Methods

### 2.1. Study Design

We performed a secondary analysis of the database of an international survey prepared and collected by the University of Maryland and the social network Facebook (Facebook, Inc., Menlo Park, CA, USA) [16]. The objective of the survey was to evaluate the sociodemographic characteristics, comorbidities, mental health, economic and food insecurity in the context of COVID-19 [16]. In addition, the survey analyzed compliance with mitigation strategies against COVID-19 and attitudes or practices related to vaccination against COVID-19 [16]. The survey was applied for the first time on 23 April 2020, and since then it has been applied daily in more than 200 countries around the world.

### 2.2. Population, Sample, and Sampling

**Figure 1:**
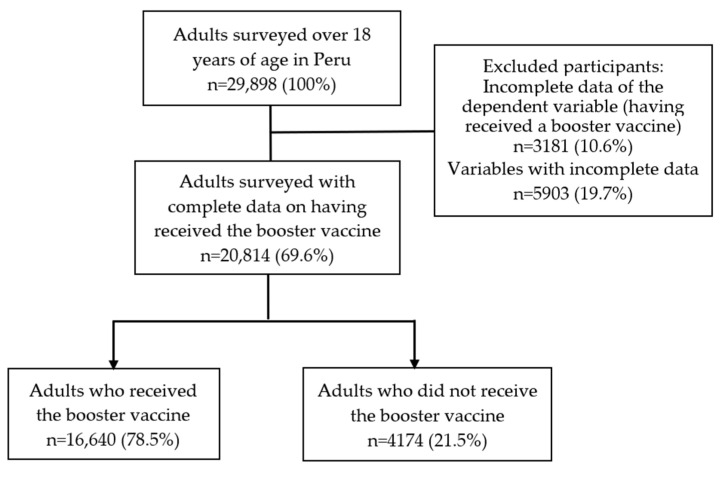
Flowchart of the selection of participants included in the analysis.

The study population included Facebook users over the age of 18. For this study, we included users residing in Peru who responded to the survey between 13 February 2022, and 14 April 2022 (60 days). Participants who did not have data regarding the self-reported of having received at least one booster dose of any COVID-19 vaccine or who did not know whether the dose they received was a booster were excluded. Likewise, we excluded answers with incomplete information in some of the variables of interest. Finally, we analyzed the data of 20,814 adults from Peru (Figure 1).

### 2.3. Sample and Sampling

Sampling was randomly stratified using administrative boundaries within countries or territories to provide geographic coverage. The selection of the participants surveyed was random and was recalculated daily within the sampling framework of all Facebook users according to geographic region and country. If a user refused to participate in the survey, Facebook would randomly invite another user within the same geographical area who had not responded to the survey in the last eight weeks. The survey was globally representative, as those users who were not eligible to participate in the survey due to geographic and language restrictions represented less than 5% globally.

### 2.4. Questionnaire

The latest version of the survey (v13, updated on 30 January 2022) has eight sections. The first section includes questions about symptoms related to COVID-19. The second section includes questions about performing diagnostic tests for COVID-19. The third section has information related to vaccination against COVID-19. The fourth section evaluates sociodemographic characteristics. The fifth section includes questions on behaviors related to COVID-19. The sixth section provides information about the perception, prevention practices and sources of information against COVID-19. The seventh section is on health conditions and information related to children. Finally, the last section includes questions about the respondent’s occupation. This survey has been used to develop previous studies [17,18,19,20,21,22] and the survey methodology has been described in more detail elsewhere [16].

### 2.5. Variables

#### 2.5.1. Outcome Variable: Not Receiving a Booster Dose against COVID-19

The study outcome was having received a booster dose of the COVID-19 vaccine, assessed by the following question: “Have you received an additional or booster dose of the COVID-19 vaccine?”. This question had four possible alternatives: “Yes, I have received an additional dose or booster shot”, “Yes, I have received two or more additional doses or booster shots”, “No, I have not received an additional dose or booster shot”, and “I don’t know”. This variable was dichotomized considering the first two alternatives as “having received the booster dose against COVID-19”, and the third alternative as “not having received the booster dose”. Those who answered not knowing if they had received a booster were excluded.

#### 2.5.2. Independent Variables

We included sociodemographic characteristics such as gender (male, female), age (18–24, 25–34, 35–44, 45–54, 55–64, 65–74, or 75 years or older), educational level (postgraduate, university, secondary or pre-university, primary or without formal education), employment (employed or not employed), and area of residence (city, town, or rural area). Regarding area of residence, in the survey, a town was considered as a populated area with fixed boundaries and a local self-government, a city as an important or large town, and a village as a group of houses and other buildings, usually in the countryside, that is smaller than a town. Likewise, we collected the variable comorbidity considering the presence or absence of the following comorbidities: asthma, chronic obstructive pulmonary disease (COPD) or chronic bronchitis or emphysema, cancer, diabetes, high blood pressure, kidney disease, compromised or weakened immune system, heart attack or other heart disease and obesity. For this variable, we considered three categories (none, one to two, and three or more comorbidities). Additionally, as a study variable we included a history of having had COVID-19 (no or yes).

### 2.6. Statistical Analysis

The databases used in this study were downloaded in text format “.txt” and were imported into the statistical package STATA v15.0 (StataCorp, College Station, TX, USA). All analyses were conducted considering the complex sampling of the survey [16] using the *svy* command. We described the variables using absolute frequencies and weighted proportions with their respective 95% confidence intervals (95%CI). We estimated confidence intervals using the transformed logit method and assessed the variance by first-order Taylor linearization. Bivariate analysis between the independent variables and not having received a booster dose against COVID-19 was performed using the Chi-square test with Rao–Scott correction. We used generalized linear models of the Poisson family with logarithmic link function to estimate the factors associated with not having received a booster dose against COVID-19. We estimated crude (cPR) and adjusted (aPR) prevalence ratios with their respective 95%CI. We used a statistical criterion to choose the variables to include in the fitted model (those with a *p* < 0.05 in the crude model). We evaluated the possible collinearity of the associated factors included in the final fitted model. Statistical significance was set at *p* < 0.05.

### 2.7. Ethical Considerations

We analyzed a secondary database that collected data without personal identifiers, and thus, approval from an institutional research ethics committee was not required. Likewise, the participants provided informed consent before starting the survey. On the other hand, access to the databases was given with the permission of the University of Maryland.

## 3. Results

### 3.1. Characteristics of the Study Sample

We analyzed a sample of 20,814 adults from Peru between 13 February and 13 April 2022. Of these, 51.3% (n = 9900) were women, 22.1% (n = 4455) were between 25 and 34 years old, 45.8% (n = 9784) had a college education, 58.5% (n = 12,655) reported being employed, and 83.2% (n = 17,991) lived in a city. In addition, 81.1% (n = 16,694) reported having no comorbidities, 50.2% (n = 10,510) had had COVID-19 at some point, and 21.5% (n = 4174) reported not having received a booster vaccine against COVID-19 (Table 1).

### 3.2. Bivariate Analysis according to Not Having Received the Booster Vaccine against COVID-19

We identified statistically significant differences in the bivariate analysis between the independent variables and not having received the COVID-19 booster vaccine. The frequency of not having received the COVID-19 booster vaccine was higher in males (*p* = 0.006), adults between 18 and 24 years old (*p* < 0.001), residents of a village or rural area (*p* < 0.001), participants with primary school or less (*p* < 0.001), with no employment (*p* = 0.002), with no comorbidities (*p* < 0.001), and with a history of COVID-19 (*p* < 0.001) (Table 2).

### 3.3. Factors Associated with Not Having Received the Booster Vaccine against COVID-19

The adjusted regression model showed a higher prevalence of not having received the COVID-19 booster vaccine among people aged 65 to 74 years (aPR = 1.04; 95%CI: 1.01–1.06; *p* = 0.002), 55 to 64 years (aPR = 1.06; 95%CI: 1.03–1.08; *p* < 0.001), 45 to 54 years (aPR = 1.10; 95%CI: 1.08–1.13; *p* < 0.001), 35 to 44 years (aPR = 1.16; 95%CI: 1.14–1.19; *p* < 0.001), 25 to 34 years old (aPR = 1.26; 95%CI: 1.21–1.30; *p* < 0.001), and 18 to 24 years old (aPR = 1.37; 95%CI: 1.33–1.41; *p* < 0.001) compared to those aged 75 and over. Compared to having a postgraduate education, having a university education (aPR = 1.03; 95%CI: 1.02–1.05; *p* < 0.001), secondary or pre-university education (aPR = 1.07; 95%CI: 1.05–1.09; *p* < 0.001), or having a primary level or no formal education (aPR = 1.11; 95%CI: 1.05–1.18; *p* < 0.001) were associated with a higher prevalence of not having received the booster vaccine. Being employed (aPR = 1.01; 95%CI: 1.00–1.02; *p* = 0.022) and having had COVID-19 (aPR = 1.03; 95%CI: 1.01–1.04; *p* < 0.001) were associated with a higher prevalence of not having received the booster shot. Compared to living in the city, living in a town (aPR = 1.05; 95%CI: 1.02–1.07; *p* = 0.001) or in a rural area (aPR = 1.06; 95%CI: 1.03–1.10; *p* = 0.001) were associated with a higher prevalence of not having received the booster vaccine against COVID-19. On the other hand, a lower prevalence of not having received the booster vaccine for COVID-19 was identified in females (aPR = 0.97; 95%CI: 0.96–0.99; *p* = 0.001) (Table 3).

## 4. Discussion

The present study identifies the factors associated with not having received the booster dose for COVID-19 in the Peruvian population. The report of not having received a booster vaccine against COVID-19 was found in two out of ten Peruvians. Sociodemographic characteristics and having had COVID-19 were related to an increased probability of not having received a booster vaccine against COVID-19, while being a female decreases this probability. This is, to our knowledge, the first study that evaluated the prevalence and factors associated to not having received the COVID-19 booster vaccine carried out in Peruvian adults.

The booster dose is part of the vaccination campaigns against COVID-19 in all Latin American countries [23]. As with the start of vaccination, booster dose vaccination campaigns began on different dates in Latin American countries, with the Dominican Republic being the first country to start this campaign in July 2021, followed by Chile and Uruguay [24]. These campaigns are complex processes in which not only is the start date relevant [25], but also structural aspects of the health systems [26], which could explain the variations in the coverage of the booster dose among the countries. For example, by 20 April 2022, the Dominican Republic had achieved booster vaccination in only 21.7% of its population, while in Chile the percentage was 87.2% and in Uruguay 65.7% [23]. In Peru, the campaign of the third dose began on 15 October 2021 and as of 25 October 2022, half the population had been covered according to the Ministry of Health (MINSA) of Peru [14]. The variation shown by our results for the frequency of the population vaccinated with the third dose is explained by the nature of the information sources, since unlike the official data from the MINSA [26], our study used a sample of users of a social network. It is not clear why people who received the booster dose were more likely to respond to a survey; however, this may be related to the empowerment of patients in decision-making regarding their disease [27]. In this sense, making their experience known could be perceived as a way of contributing to knowledge of the disease.

In Peru, the vaccination strategy with the booster dose included the entire population over 18 years of age in whom at least three months had elapsed since the application of the second dose [28]. It began in risk groups for COVID-19, such as elderly patients [29], as in other countries on the continent [30], which could explain our findings. As of 26 April 2022, the MINSA reported that booster dose coverage was greater than 90% in people over 70 years of age in almost all the regions of Peru [14], while in younger age groups the percentage of vaccination coverage differed [14].

A person from a rural area was less likely to have a booster dose for COVID-19, which is consistent with the intention to be vaccinated against COVID-19 in Peru. Previously, it was described that living in a town, village, or rural area was associated with a lower vaccination intention compared to people living in the city [19]. These results suggest that vaccination campaigns against the disease should focus on rural areas, considering the impact that the pandemic has had on this population. Peru has been one of the countries most affected by the pandemic for reasons including problems due to its fragmented and centralized health system and in decision-making regarding the treatment of infected patients [31,32]. Although there are no studies that verify the distribution of infected patients according to whether they live in a rural or urban area, some studies have shown while the jungle region of Peru did not necessarily have the highest number of infected [13], it had the highest mortality from COVID-19 [33], and some of its departments had the highest percentages of excess mortality in our country [34]. This is relevant because in the departments of the jungle, such as Amazonas, there is a mostly rural population according to the 2017 census (58.5%) [35]. Although we cannot determine the reasons why rural residents had a lower proportion of having received a booster dose for COVID-19, it is likely that characteristics such as educational level and level of employment and difficult access to health care in this region play a role. In that sense, we found that any educational level below the postgraduate level was associated with a lower prevalence of not having received a booster dose. Coincidentally, in Peru, rural areas show worse indicators at all educational levels compared to urban populations [36]. The crude and net school enrollment rate of the population aged 12 to 16 is lower in the rural population [36]. Likewise, a higher average number of years of study is observed in residents of urban areas than in rural areas, with a gap of 3.8 years of study between residents of both areas [36]. Regarding higher education, only 2.6% of the rural population achieves this compared to 20.5% of the urban population [36]. Additionally, these areas show worse employment indicators than urban areas. Although the working-age population decreased by 0.7% in rural areas, unlike the urban population that increased by 2.2% from 2007 to 2017, the economically active population in rural areas decreased by 0.5% in 2017 and increased by 2.5% in the urban population [37]. These results emphasize the need for strategies to promote the booster dose to include rural populations, since they constitute a high-risk population subgroup due to the economic impact of the disease.

A noteworthy finding is that a history of having had COVID-19 increased the probability of not having received a booster dose for this disease. Previously in Peru, having had symptoms of this disease and the fear of a family member becoming infected with COVID-19 was associated with the intention to be vaccinated, presumably associated with fear of the disease and its effects [19]. It is possible that the perception that a booster dose is not necessary after having previously had the infection is one of the reasons, as shown in previous studies [38]. Likewise, it is likely that the time elapsed since the beginning of the pandemic has exhausted the interest in maintaining biosafety measures, a phenomenon known as “pandemic fatigue”, leading people to reduce their adherence to community mitigation measures [39,40], including the receipt of booster doses.

Previously, it was described that women had less intention to be vaccinated against COVID-19 in Peru [19]. On the contrary, the findings of our study indicate that being a woman decreased the probability of not having received the booster dose. Although “pandemic fatigue” is described as affecting more women than men [41], who are more exposed to anti-vaccine groups and who are more susceptible to conspiracy theories related to the disease [42,43,44], in Peru, men showed the highest excess mortality [34]. Although it is possible that fear of the disease and its effects influence this decision, in Latin America there are gender aspects that influence mental health problems and adherence to community mitigation measures not evaluated in our study that may also play a role [17] and might explain this result.

## 5. Limitations

Our study has some limitations. In the first place, since the respondents were users of an online social network, information was only obtained from people with access to the internet and social networks, which could vary between the different regions of the country or rural population. Second, the variables included, and their definitions, are subject to the pre-established definition of the parent survey, and there may be relevant variables not included in the analysis due to their unavailability. Third, the data was obtained by self-reporting, which could produce underreporting of information. Fourth, causality among the variables evaluated cannot be established due to the study design. Fifth, we do not have the non-response rate, which is relevant in the context of an online survey, and thus our results could be biased due to the rate of rejection of users in the survey, as well as the possibility of occurrence of voluntary response bias. Despite these limitations, a study with a large sample with national representativeness can help understand the subject of study.

## 6. Conclusions

In conclusion, being under 75 years of age, having less than a graduate level of education, not having a job, living in a town or village/rural area, and having had COVID-19 increased the probability of not having received the booster dose. On the other hand, being female decreased the probability of not having received the booster dose. This is, to our knowledge, the first study in Peruvian adults and in Latin American region that evaluated the prevalence and factors associated with not having received the COVID-19 booster vaccine. Several studies focused on the prevalence and factors associated with vaccination intention hesitancy and refusal, however, our study assessed adults that reported having received booster vaccine against COVID-19. Our results identify population groups in which vaccination campaigns related to the third dose should have an impact. Although the number of cases has decreased in our country [13], considering that almost half of the Peruvian population has still not received this booster [14] and that the campaign of the fourth dose has begun [45], completion of the vaccination schedule with the booster dose will allow us to be prepared for the effects of possible new variants of concern [8].

## Figures and Tables

**Table 1:** Characteristics of the study sample (n = 20,814).

Characteristics	n	% *	95%CI *
Gender			
Male	10,914	48.7	44.8–52.7
Female	9900	51.3	47.3–55.2
Age (years)			
18–24	3458	15.8	13.8–18.1
25–34	4455	22.1	21.2–23.0
35–44	4175	20.0	19.1–20.9
45–54	3933	18.6	18.0–19.3
55–64	3070	12.2	11.4–13.1
65–74	1478	9.9	8.5–11.5
75 years or older	245	1.4	0.9–2.1
Area of residence			
City	17,991	83.2	73.0–90.1
Town	1721	10.0	5.8–16.7
Village or rural area	1102	6.8	4.2–10.8
Educational level			
Primary school or less	341	2.1	1.7–2.7
Secondary school—pre-university	7713	39.1	37.0–41.3
University	9784	45.8	44.2–47.4
Postgraduate	2976	12.9	12.0–13.9
Employment			
No	8159	41.5	40.4–42.6
Yes	12,655	58.5	57.4–59.6
Comorbidities			
None	16,694	81.1	78.8–83.2
1 to 2	3785	17.2	15.4–19.2
Greater than or equal to 3	335	1.6	1.3–2.1
History of COVID-19			
No	10,304	49.8	48.5–51.1
Yes	10,510	50.2	48.9–51.5
Having received a booster			
Yes	16,640	78.5	74.8–81.7
No	4174	21.5	18.3–25.2

95%CI: 95% confidence intervals. * Weighted percentages according to survey complex sampling.

**Table 2:** Prevalence of not having received the booster dose according to the characteristics of the study sample.

Characteristics	Not having Received Booster						
	Yes			No			*p*-Value **
	n	% *	95%CI *	n	% *	95%CI *	
Gender							**0.006**
Male	8785	77.1	73.0–80.7	2129	22.9	19.3–27.0	
Female	7855	79.7	76.3–82.8	2045	20.3	17.2–23.7	
Age (years)							**<0.001**
18–24	2018	56.9	54.8–58.9	1440	43.1	41.1–45.2	
25–34	3262	70.2	64.3–75.6	1193	29.8	24.4–35.7	
35–44	3411	79.9	76.0–83.2	764	20.1	16.8–24.0	
45–54	3459	86.6	83.8–88.9	474	13.4	11.1–16.2	
55–64	2850	91.5	88.3–93.8	220	8.5	6.2–11.7	
65–74	1402	94.5	92.5–95.9	76	5.5	4.1–7.5	
75 years or older	238	97.7	93.9–99.2	7	2.3	0.8–6.1	
Area of residence							**<0.001**
City	14,628	80.2	77.1–82.9	3363	19.8	17.1–22.9	
Town	1230	70.3	67.3–73.1	491	29.7	26.9–32.7	
Village or rural area	782	69.5	63.6–74.8	320	30.5	25.2–36.4	
Educational level							**<0.001**
Primary school or less	229	70.1	64.8–74.9	112	29.9	25.1–35.2	
Secondary school—pre-university	5747	73.1	69.5–76.5	1966	26.9	23.5–30.5	
University	7967	80.3	76.1–83.9	1817	19.7	16.1–23.9	
Postgraduate	2697	89.5	85.5–92.6	279	10.5	7.4–14.5	
Employment							**0.002**
No	6362	77.1	72.9–80.9	1797	22.9	19.1–27.1	
Yes	10,278	79.4	75.9–82.4	2377	20.6	17.6–24.1	
Comorbidities							**<0.001**
None	13,181	77.5	73.6–80.9	3513	22.5	19.1–26.4	
1 to 2	3164	82.3	79.4–84.8	621	17.7	15.2–20.6	
Greater than or equal to 3	295	87.5	83.5–90.6	40	12.5	9.4–16.5	
History of COVID-19							**<0.001**
No	8515	80.8	76.8–84.2	1789	19.2	15.8–23.2	
Yes	8125	76.1	72.5–79.4	2385	23.9	20.6–27.5	

95%CI: 95% confidence interval. * Weighted percentages according to survey complex sampling. ** Calculated by Chi2 test of independence with Rao–Scott correction for complex sampling. *p*-values < 0.05 are in bold.

**Table 3:** Factors associated with not having received a booster dose of the COVID-19 vaccine.

Characteristics	Crude Model			Adjusted Model		
	cPR	95%CI	*p*-Value	aPR	95%CI	*p*-Value
Gender						
Male	Ref.			Ref.		
Female	0.98	0.96–0.99	**0.008**	0.97	0.96–0.99	**0.001**
Non-binary						
Age (years)	Ref.			Ref.		
75 years or older	1.03	1.01–1.05	**0.004**	1.04	1.01–1.06	**0.002**
65–74	1.06	1.03–1.09	**<0.001**	1.06	1.03–1.08	**<0.001**
55–64	1.11	1.09–1.13	**<0.001**	1.10	1.08–1.13	**<0.001**
45–54	1.17	1.15–1.20	**<0.001**	1.16	1.14–1.19	**<0.001**
35–44	1.27	1.22–1.31	**<0.001**	1.26	1.21–1.30	**<0.001**
25–34	1.40	1.36–1.44	**<0.001**	1.37	1.33–1.41	**<0.001**
Area of residence						
City	Ref.			Ref.		
Town	1.08	1.05–1.11	**<0.001**	1.05	1.02–1.07	**0.001**
Village or rural area	1.09	1.05–1.12	**<0.001**	1.06	1.03–1.10	**0.001**
Educational level						
Postgraduate	Ref.			Ref.		
University	1.08	1.07–1.10	**<0.001**	1.03	1.02–1.05	**<0.001**
Secondary school—pre-university	1.15	1.13–1.17	**<0.001**	1.07	1.05–1.09	**<0.001**
Primary school or less	1.18	1.12–1.24	**<0.001**	1.11	1.05–1.18	**<0.001**
Employment						
Yes	Ref.			Ref.		
No	1.02	1.01–1.03	**0.004**	1.01	1.00–1.02	**0.022**
Comorbidities						
Greater than or equal to 3	Ref.			Ref.		
1 to 2	1.05	1.02–1.08	**0.004**	1.01	0.97–1.05	**0.562**
None	1.09	1.06–1.12	**<0.001**	1.02	0.98–1.06	**0.327**
History of COVID-19						
No	Ref.			Ref.		
Yes	1.04	1.03–1.05	**<0.001**	1.03	1.01–1.04	**<0.001**

cPR: crude prevalence ratio; aPR: adjusted prevalence ratio; 95%CI: 95% confidence interval. Prevalence ratios and confidence intervals were calculated considering the complex sampling of the survey. *p*-values < 0.05 are in bold.

## Data Availability

Restrictions apply to the availability of these data. Authors obtained the data after signing a contract with the University of Maryland and have not permission to share the database.
